# Hurricanes, El Niño and harmful algal blooms in two sub-tropical Florida estuaries: Direct and indirect impacts

**DOI:** 10.1038/s41598-020-58771-4

**Published:** 2020-02-05

**Authors:** Edward J. Phlips, Susan Badylak, Natalie G. Nelson, Karl E. Havens

**Affiliations:** 1 Fisheries and Aquatic Sciences Program, SFRC, University of Florida, Gainesville, Florida USA; 2 Biological and Agricultural Engineering, North Carolina State University, Raleigh, North Carolina USA; 3 Center for Geospatial Analytics, North Carolina State University, Raleigh, North Carolina USA; 4 Florida Sea Grant, University of Florida, Gainesville, Florida USA

**Keywords:** Phenology, Marine biology

## Abstract

Future increases in the intensity of hurricanes and El Niño periods predicted by climate change models have focused attention on their role in stimulating harmful algal blooms (HABs). A series of hurricanes that recently impacted Florida (USA) provided a unique opportunity to explore the relationships between hurricanes, El Niño and HABs in two Florida estuaries subject to repeated intense ecosystem disruptive HABs, the Indian River Lagoon and the St. Lucie Estuary. The roles that hurricanes and El Niño play in contributing to HAB events are examined in the context of key structural and functional features of each estuary and their watersheds, including morphology, water residence time and hydrology, such as the influence of Lake Okeechobee discharges into the St. Lucie Estuary. The most direct impact was the increase in rainfall associated with hurricanes and El Niño, resulting in enhanced nutrient loads which drive HABs in the Indian River Lagoon and Lake Okeechobee. Major HABs in Lake Okeechobee also present an indirect threat of freshwater HAB blooms in the St. Lucie Estuary via mandated discharges from the lake into the estuary during high rainfall periods. Conversely, during the absence of HABs in Lake Okeechobee, short water residence times produced by discharges into the St. Lucie Estuary can result in lower bloom intensities.

## Introduction

There is a consensus about the role that human activity plays in nutrient enrichment of aquatic environments as drivers of harmful algal blooms (HABs)^1,2^. There are also growing concerns that anticipated future changes in climatic conditions will increase threats for HABs^2,3^. Among these climatic threats are increases in the intensity and duration of hurricanes and El Niño periods^4–6^. High rainfall and winds associated with storms and elevated rain during El Niño periods in certain regions of the world can impact a range of processes relevant to phytoplankton dynamics and HAB development, including nutrient loads, physical disruption of ecosystems and ecosystem flushing rates^7–10^. These impacts can be exacerbated by human influences on nutrient loads and hydrology^11,12^.

A series of hurricanes that recently impacted the peninsula of Florida, and the long record of El Niño/La Niña cycles, provide an opportunity to explore the potential impacts on HABs. The effects of storm and El Niño-driven changes to the hydrology and nutrient status of coastal estuaries can be difficult to define without a basic understanding of the structure and function of both the core estuary and its watershed. For example, storm enhanced watershed runoff can increase external nutrient loads that fuel algal blooms, or contain high HAB biomass from freshwater ecosystems in the watershed, or conversely, in some ecosystems elevated flushing rates can limit the intensity of autochthonous HABs by reducing water residence times. In addition to bringing high rainfall to impacted areas which enhance nutrient loads, the winds associated with storms can cause physical damage (e.g. erosion) or disruption (e.g. sediment re-suspension) of aquatic ecosystems, contributing to internal nutrient loading and re-distribution of nutrient sources and sinks^13,14^.

**Figure 1:**
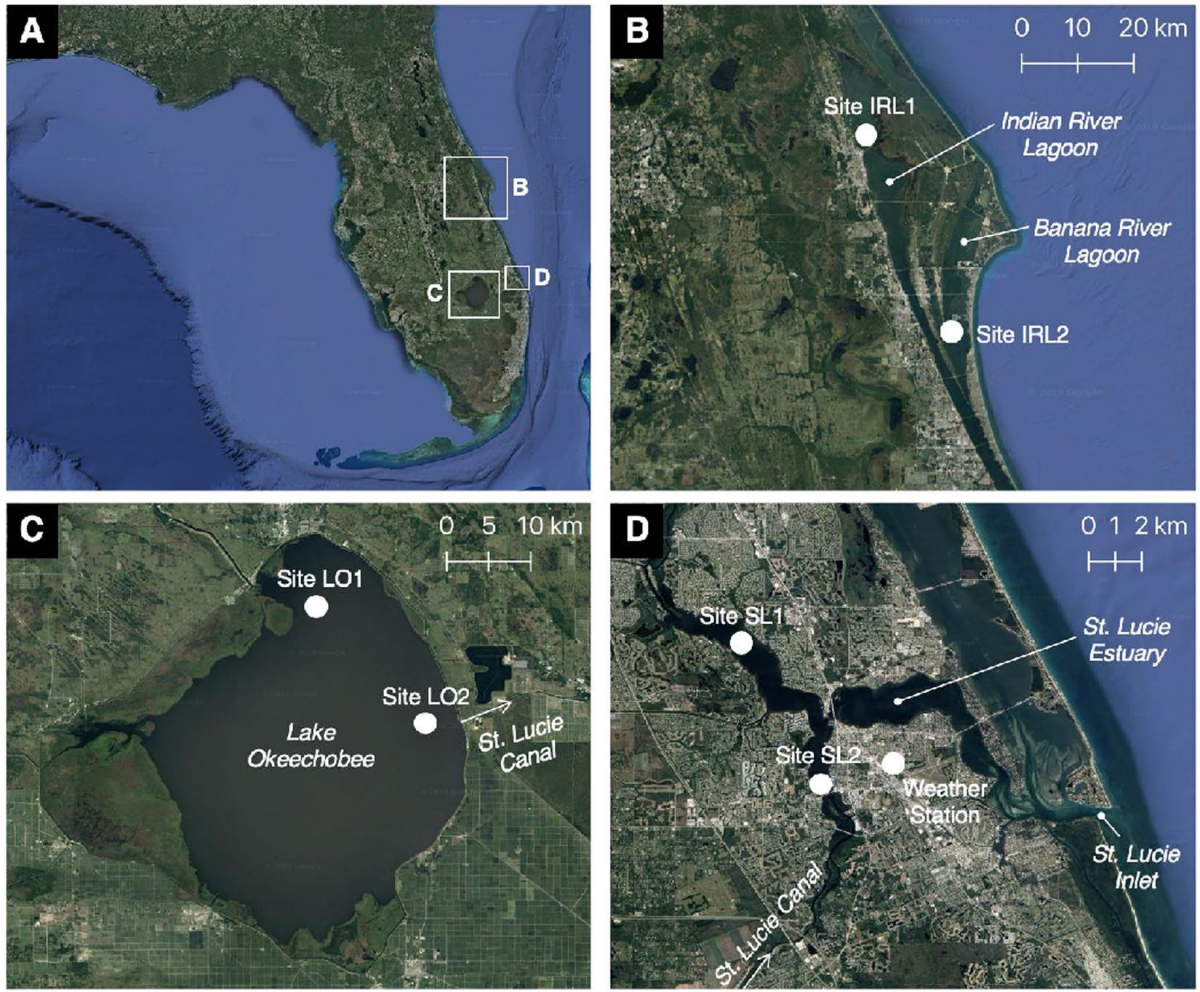
Sampling site maps for the northern Indian River Lagoon (**B**), including the connected Banana River lagoon component, Lake Okeechobee (**C**) and the St. Lucie Estuary (**D**). The top left panel (**A**) shows the location of the three ecosystems in the peninsula of Florida. Maps created using QGIS 3.2.3 with imagery from Google Maps using the XYZ Tiles service. Imagery data providers include LDEO-Columbia, NSF, NOAA, U.S. Navy, NGA, GEBCO, INEGI, SIO, Landsat/Copernicus).

**Figure 2:**
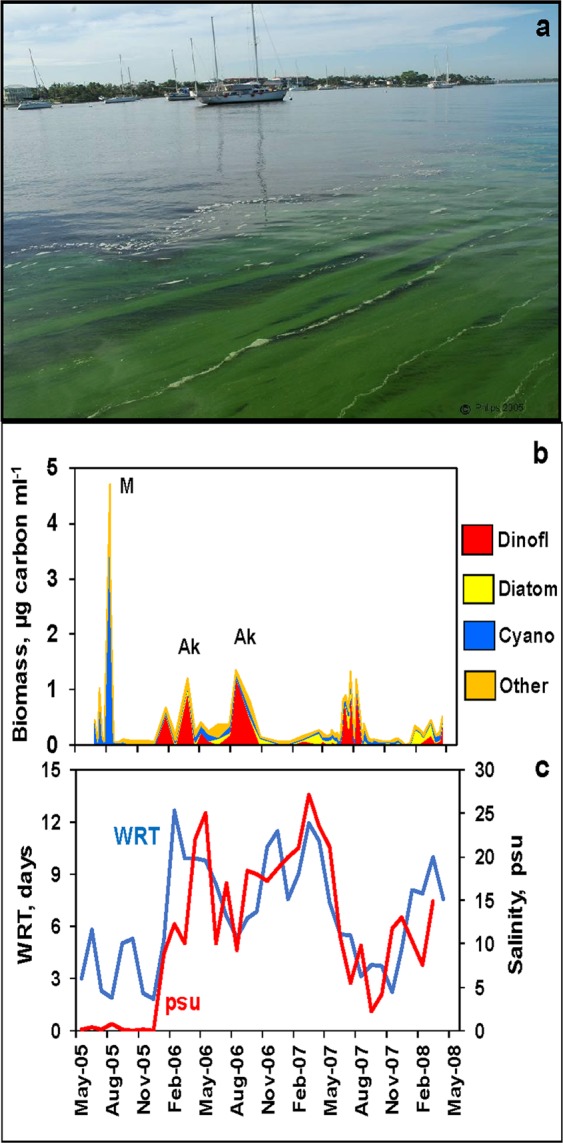
Image of major cyanobacteria bloom in the St. Lucie Estuary in August 2005 (**a**, photo by author E. J. Phlips), phytoplankton biomass time series at Site SL2 in the South Fork of the estuary, divided into four major groupings, i.e. dinoflagellates, diatoms, cyanobacteria and “other” (letters above major bloom indicate dominant species, A – marine dinoflagellate *Akashiwo sanguinea;* M – freshwater cyanobacterium *Microcystis aeruginosa*) (**b**). Salinities (psu) and water residence times for the inner estuary (**c**).

We address these issues and examine the relationships between hurricanes (and more generally tropical cyclones), El Niño periods and HABs in two sub-tropical ecosystems, the St. Lucie Estuary and the Indian River Lagoon (Fig. 1). In subtropical ecosystems, relatively modest seasonal variability in temperature and irradiance can reduce the predictability of seasonal trends in phytoplankton biomass and composition^15^. Consequently, other variables can take on greater importance in driving phytoplankton dynamics, often on longer and less predictable time intervals^16^. Two such factors are multi-year cyclical patterns (e.g. El Niño/La Niña cycles) and stochastic variability in rainfall intensity and wind associated with storm events. Both the St. Lucie Estuary and Indian River Lagoon have experienced significant hurricane activity, but each illustrates a different set of drivers and consequences associated with storm events as it relates to HABs. In the St. Lucie Estuary, hurricanes can indirectly impact HABs by increasing the potential for introduction of high algae biomass from freshwater systems in the watershed (Fig. 2a). In the Indian River Lagoon, both hurricanes and El Niño periods have a direct positive effect on HABs of internal origin (i.e. autochthonous), predominantly through the enhancement of nutrient loads. These relationships are explored within the context of key structural and functional features of each estuary and its watershed, including water residence times, composition of dominant algal species during HABs within and entering the estuary from watersheds and temporal patterns of hydrologic management activities, such as regulated discharges from Lake Okeechobee into the St. Lucie Estuary via the St. Lucie Canal^17,18^.

## Methods

### Site description

In the St. Lucie Estuary, the study focused on two sampling sites in the inner regions of the St. Lucie Estuary, Site SL1 in the North Fork region and Site SL2 in the South Fork region (Fig. 1). The St. Lucie Estuary has an area of 29 km^2^ and is shallow throughout (i.e. mean depth 2–2.5 m)^19,20^. In addition to two natural inflows, Ten-mile Creek and Old South Fork, three man-made canals (C-23, C-24 and C-44) were added to the system in the first half of the 20^th^ Century to control hydrology in the region, including the regulation of water releases from Lake Okeechobee into the St. Lucie Canal (C-44) for flood control (Fig. 1). The canals provide an average of 75% of the freshwater discharge into the estuary, and all the inflows are managed by means of water control structures, such as locks, dams and water pumping stations^21^. The shallow depth and relatively small size of the St. Lucie Estuary result in rapid and spatially extensive responses to changes in discharge^22^. Water residence times in the South Fork/North Fork region of the estuary range from 1–16.5 days, based on CH3D hydrodynamic/salinity models^19^. Salinity isoclines can move substantial distances up and down the estuary on time scales of days to weeks, and vertical stratification is generally short-lived. The estuary is microtidal (amplitude <0.5 m)^22^.

Lake Okeechobee, located in south-central Florida (27°00′ N, 80°50′ W), is the largest lake in the southeast United States (1,730 km^2^ surface area). It is shallow (2.7 m mean depth), eutrophic and frequently subject to intense cyanobacteria blooms^17^. It has been impacted repeatedly by hurricanes, sometimes several in the same year. The lake has distinct zones that differ in their ecological structure and function, including a large comparatively deep (i.e. 4–5 m) central zone characterized by flocculent muddy sediments, a shallower (<3 m) perimeter zone characterized by firmer sediments, and a shallow northern perimeter zone subject to the largest external inflows from the water shed^23,24^. In terms of HAB events, blooms often begin in the shallow perimeter regions of the lake because of higher light availability and proximity to external nutrient inputs, but can spread throughout the lake^24^. In order to capture potential variability in conditions, data for two sites were included in these analyses, i.e. Sites LO1 and LO2 in the nearshore region of the lake, near the outflow to the St. Lucie Canal, which flows into the St. Lucie Estuary (Fig. 1).

In the Indian River Lagoon, the study focused on two sampling sites located in two separate sub-basins of the northern Indian River Lagoon subject to frequent HABs: Site IRL1 in the northern Indian River Lagoon near Titusville, and Site IRL2 in the central Banana River Lagoon (Fig. 1). Both sub-basins are microtidal and have long water residence times, with estimated mean water half-lives (i.e. 50% exchange) of 107 days in the northern Indian River Lagoon and 156 days in the central Banana River Lagoon^25,26^. Mean water depths are approximately 2 m in both regions. The sub-basins associated with Sites IRL1 and IRL2 are both characterized by small watersheds (i.e. 35,446 ha and 5628 ha, respectively), relative to the size of the receiving basins (i.e. 16,465 ha and 10,202 ha, respectively), but their watersheds differ in terms of percent distribution of land-uses^27,28^. The sub-basin of Site IRL1 had 65% undeveloped, 24% agricultural and 11% urban/residential land-use areas in 2009^28^. The sub-basin of Site IRL2 had significantly higher urban/residential area at 65%, low agricultural (4%) and 33% undeveloped land-use areas in 2009^28^.

### Sampling and field collections

Sites SL1 and SL2 in the St. Lucie Estuary were sampled on a weekly basis from May 2005 through April 2008. Sites IRL1 and IRL2 in the northern Indian River Lagoon were sampled monthly from September 1997 to August 2005, and twice monthly from September 2005 through April 2018. Temperature and salinity were measured at the surface and near the bottom at each site with a Hydrolab Quanta environmental multi probe. Water samples for phytoplankton analysis were collected with a vertical integrating sampling tube that captured water from the surface to within 0.1 m of the bottom, to avoid sample bias resulting from vertical stratification of phytoplankton. Duplicate aliquots were preserved on site, one with Lugol’s solution and one with buffered glutaraldehyde.

### Nutrient and chlorophyll a data

Monthly total Kjeldahl nitrogen and total phosphorus data for Indian River Lagoon (1997–2018) were obtained from the St. Johns River Water Management District (Palatka, Florida). Nitrate, soluble reactive phosphorus, total suspended solids and chlorophyll *a* data for the two sites in Lake Okeechobee (2004–2007) were obtained from the South Florida Water Management District (West Palm Beach, Florida).

### Climate, discharge and remotely-sensed cyanobacteria observations

Rainfall data for the Stuart and Titusville (Florida) meteorological stations were obtained from the NOAA Climatological Data for Florida web site (www.ncdc.noaa.gov/IPS). Flow data capturing discharge from Lake Okeechobee into the St. Lucie Canal (Site 02276877) were obtained from the U.S. Geological Survey. Satellite imagery captured by MERIS and Sentinel-3 OLCI were analyzed using the Cyanobacteria Index (CI)^29^ for cyanobacteria abundance and distribution in Lake Okeechobee. The CI was calculated from MERIS imagery for dates in 2005, and Sentinel-3 imagery in 2018.

### Plankton analysis

General phytoplankton composition was determined using the Utermöhl method^30^. Samples preserved in Lugol’s were settled in 19-mm diameter cylindrical chambers. Phytoplankton cells were identified and counted at 400× and 100× with a Leica phase contrast inverted microscope. At 400×, a minimum of 100 cells of a single taxon and 30 grids were counted. If 100 cells of a single taxon were not counted by 30 grids, up to a maximum of 100 grids were counted until 100 cells of a single taxon were reached. At 100×, a total bottom count was completed for taxa >30 µm in size.

Fluorescence microscopy was used to enumerate picoplanktonic cyanobacteria (e.g. *Synechococcus*.spp., spherical picocyanobacteria spp.) at 1000× magnification^31^. Subsamples of seawater were filtered onto 0.2 µm Nuclepore filters and mounted between a microscope slide and cover slip with immersion oil. If not analyzed immediately, samples preserved with buffered glutaraldehyde were refrigerated and counted within 72 h.

Cell biovolumes were estimated by assigning combinations of geometric shapes to fit the characteristics of individual taxa^32,33^. Specific phytoplankton dimensions were measured for at least 30 randomly selected cells. Species which vary substantially in size, such as many diatom species, were placed into size categories. Phytoplankton biomass as carbon values (i.e. µg carbon ml^−1^) were estimated by using conversion factors for different taxonomic groups applied to biovolume estimates: i.e. 0.065× biovolume of diatoms, 0.22× biovolume of cyanobacteria or nanoplanktonic eukaryotes, and 0.16× biovolume of dinoflagellates or other taxa^34–38^.

### Statistical and modeling methods

Basic statistical procedures (i.e. determination of mean values, standard deviations, and Spearman’s comparison of means) were carried out using SAS v9.2 (SAS Institute, Cary, North Carolina, USA).

Water residence times are expressed as E_60_ values (i.e. time in days for 60% water exchange), otherwise referred to as the e-folding time. Water residence time data for the inner St. Lucie Estuary used in this paper were provided by D. Sun of the South Florida Water Management District (W. Palm Beach, Florida). The St. Lucie Estuary is strongly influenced by tidal water exchange rates as well as freshwater flushing rates, therefore both factors are incorporated into the model formulations of water residence time^19,20^. Water residence time estimates for the study period were based on linear regression relationships developed between historical E_60_ values derived from a hydrodynamic model for 1997–1999^19,20^ and rainfall integrated over a period of two weeks prior to the date (i.e. X) of the E_60_ model estimate. Regression for North Fork was E_60_ = −0.2534X + 17.028, R^2^ = 0.77. Regression for South Fork was E_60_ = −0.0257X + 2.9239, R^2^ = 0.21. A number of factors contribute to the lower R^2^ of the relationship for South Fork, including the morphology of the estuary and the implications for tidal mixing, very shallow mean depth, small volume compared to North Fork and direct impacts of the flow-regulated discharges from the St. Lucie Canal to South Fork.

## Results and discussion

### St. Lucie Estuary-Lake Okeechobee connection

**Figure 3:**
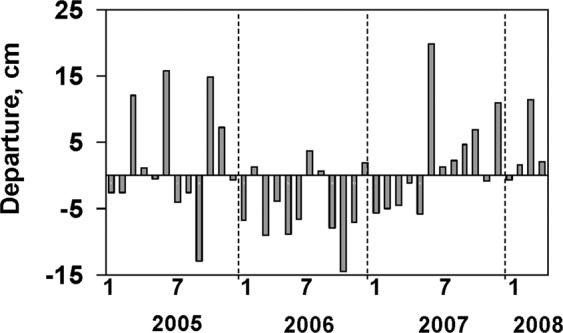
Departure from long-term average monthly rainfall at a weather station in the region of the St. Lucie Estuary at the NOAA meteorological station at Stuart, Florida (www.ncdc.noaa.gov/IPS).

The results of a three-year study of the St. Lucie Estuary provide insights into two different ways storms affect HABs, i.e. internal blooms (i.e. autochthonous) of marine species and externally introduced blooms (i.e. allochthonous) of freshwater species^39^. The largest biomass peaks of marine species were observed in late summer/early fall (August-October) of 2006 (Fig. 2b, Supplemental Fig. S1), when salinities and water residence times were comparatively high (Fig. 2c) due to below average rainfall levels (Fig. 3), providing the conditions favorable for accumulation of marine phytoplankton biomass. Millie *et al*.^40^ made a similar observation during a one-year study coinciding with a drought period in 2000, when diatom blooms were observed in the inner estuary. Conversely, periods of high rainfall, such as late summer/early fall of 2007, coincided with shorter water residence times, low salinities (Fig. 2c) and lower phytoplankton biomass (Fig. 2b), despite the fact that average nutrient levels during the 2006 period (i.e. TP, 0.20 mg L^−1^; TN, 0.90 mg L^−1^) were lower than during the same period in 2007 (i.e. TP, 0.25 mg L^−1^; TN, 1.20 mg L^−1^). These observations indicate that periods of high rainfall and discharge from the watershed can restrict marine phytoplankton biomass due to diminished water residence times^39^, highlighting the potential importance of water residence time in modifying the potential intensity of HABs involving marine species. The impact of water residence time can be compounded by elevated colored dissolved organic matter and tripton (i.e. non-algal particulate matter) in watershed discharge, which can reduce light availability in the water column for phytoplankton. This is reflected by lower mean CDOM and turbidity values in the August-October period of 2006 (i.e. 59 Pt-Co and 7.8 Ntu), than the same period in 2007 (i.e. 127 Pt-Co and 18.5 Ntu). However, the magnitude of potential light limitation for phytoplankton production may be partially mitigated by the shallow mean depths in the St. Lucie Estuary (i.e. mean depths of 2–2.5 m).

**Figure 4:**
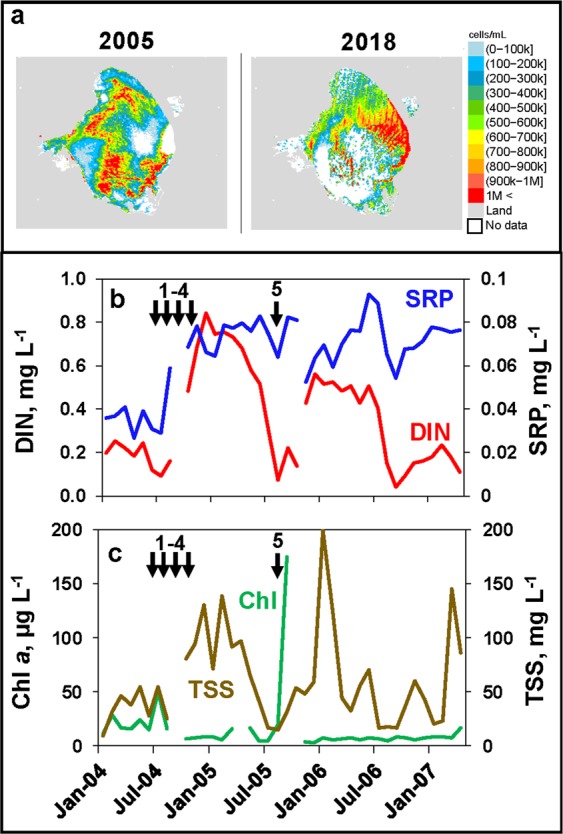
Cyanobacteria concentrations derived from weekly satellite imagery composites of Lake Okeechobee in 2005 (August 13–26) and 2018 (July 1–7) during periods of cyanobacteria blooms in the St. Lucie Estuary (**a**). Dissolved inorganic nitrogen and soluble reactive phosphorus (**b**), chlorophyll *a* and total suspended solids (**c**) concentrations in Lake Okeechobee. Values are means for Sites LO1 and LO2. Arrows indicate the timing of hurricanes that affected south and central Florida in 2004 and 2005.

**Figure 5:**
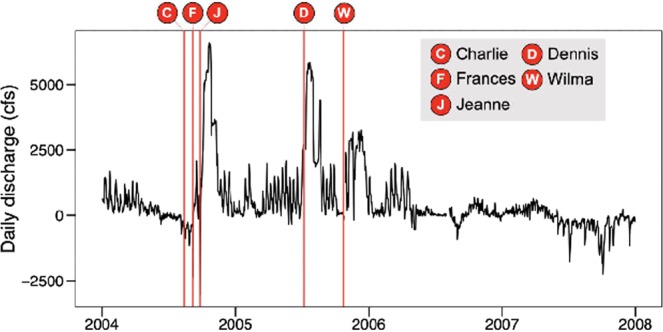
Daily discharge rates from Lake Okeechobee via the St. Lucie Canal. The timing of five hurricane events that impacted the Like Okeechobee region in 2004 and 2005 are shown on the figure.

In contrast to the relationships described for blooms of marine species, high rainfall periods can be associated with freshwater HAB events in the St. Lucie Estuary, when discharges from Lake Okeechobee into the St. Lucie Estuary via the St. Lucie Canal occur during major HAB events in the lake. The latter scenario was observed in 2005 (Fig. 4a, Supplemental Fig. S2), during a period (2004–2005) when central Florida was impacted by five hurricanes, i.e. Charley, Francis, and Jeanne in 2004, and Dennis and Wilma in 2005^39,41,42^ (Fig. 4b, Fig. 5). The storms affected Lake Okeechobee in several important ways relevant to the dynamics of HABs^17,18^. Exceptionally high rainfall resulted in large inflows of nutrient-rich water to the lake from its watershed, as evidenced by large increases in dissolved inorganic nitrogen and soluble reactive phosphorus in the lake (Fig. 4b). The increases in dissolved inorganic nitrogen may have been particularly important since the lake is more prone to nitrogen-limiting conditions for phytoplankton production than phosphorus limitation^43,44^. High winds associated with several of the hurricanes in 2004 caused intense re-suspension of muddy flocculent lake bottom sediments, resulting in high total suspended solids concentrations (Fig. 4c) and low light availability for primary production (i.e. Secchi depths less than 30 cm), as well as a potential for introduction of additional nutrients associated with bottom sediments^39,45,46^. The response of the phytoplankton community to the enhanced inorganic nutrient concentrations was not realized until the summer of 2005 (Fig. 4c), when total suspended solids concentrations declined significantly, providing the additional light necessary to support high phytoplankton production and biomass^17,44,47^. A major lake-wide bloom was observed on the lake in July-August 2005, as evidenced by satellite imagery (Fig. 4a), coinciding with a rapid decline in DIN (Fig. 4b), which reflects the high demand for inorganic nitrogen during blooms (Fig. 4b).

During the major 2005 HAB event in Lake Okeechobee, water levels in the lake were high due to excessive rainfall from the multiple hurricane events. Unlike most natural lake ecosystems, Lake Okeechobee is entirely contained within a man-made dike (i.e. Hoover Dike), built in the early 1900’s to prevent flooding in south Florida^48^. The U.S. Army Corps of Engineers is tasked with maintaining specific water levels in the lake to avoid breaching of the dike^49^. However, as an ecosystem with restricted outflows, Lake Okeechobee is characterized by long water residence times, i.e. 3.5 years^50^, which enhance the potential for intense HABs, particularly during periods of high external nutrient loads. In the summer of 2005, water levels reached a critical threshold, mandating large releases of water into the St. Lucie Canal, which discharges into the South Fork region of the St. Lucie Estuary (Fig. 1). Discharge rates went up significantly following the hurricane events (Fig. 5)^51^. The high releases in July and August 2005 coincided with a major cyanobacteria bloom in the lake (Fig. 4a), resulting in large influxes of cyanobacteria biomass into the estuary, as evidenced by the cyanobacteria peak in the St. Lucie Estuary (Fig. 2a,b). The biomass was dominated by the toxic freshwater species *Microcystis aeruginosa*, with peak average chlorophyll *a* concentrations observed in the water column of 166 µg L^−1^, and peak surface scum layer values up to 2,863 µg L^−1^ ^39^. The cyanobacteria bloom was also associated with concentrations of the hepatotoxin microcystin in excess of 1,000 µg L^−1^ in surface water grab samples^39^, which greatly exceed the World Health Organization guidelines for drinking water and recreational exposure, i.e. 1 µg L^−1^ and 10 µg L^−1^ microcystin, respectively^52,53^. During the discharge period, salinities in the inner half of the estuary were near freshwater levels (Fig. 2c), providing an environment conducive to the survival and continued growth of the toxic algae. The relationship between freshwater discharges from Lake Okeechobee and *Microcystis aeruginosa* blooms in the St. Lucie Estuary highlights how hurricanes can indirectly increase freshwater bloom potential in estuaries with strong connections to human-impacted eutrophic freshwater systems.

**Figure 6:**
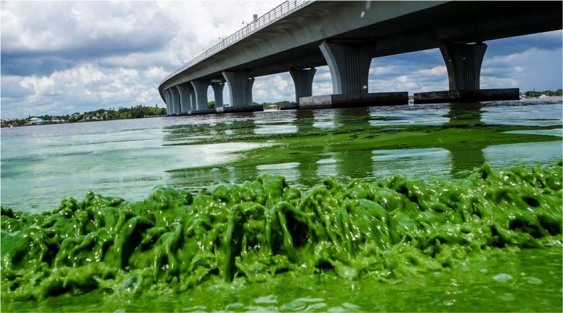
HAB picture of *Microcystis aeruginosa* bloom in the St. Lucie Estuary in 2016 (All Rights Reserved, © p77/ZUMA Press).

Intense toxic freshwater cyanobacteria blooms in the St. Lucie Estuary associated with federally-mandated flood control discharges from Lake Okeechobee have been a recurring phenomenon^17,18,39,45,54^. Hurricanes enhance the potential for blooms by elevating nutrient loads to the lake from the watersheds north and west of the lake, which in combination with long water residence times and mandated discharge from the lake, create a “perfect storm” of conditions for the potential introduction of intense HABs into the estuary. Most recently these conditions have led to re-occurrence of intense cyanobacteria blooms in the St. Lucie estuary in 2016 (Fig. 6) and 2018^18,45,54^. As in 2005, both bloom events occurred during a three-year period of strong tropical storm activity in the Lake Okeechobee region, including hurricanes Joaquin and Erika in 2015, Colin, Julia and Mathew in 2016, and Emily and Irma in 2017. The HAB event in Lake Okeechobee in 2018 is shown in a satellite image of Lake Okeechobee taken during the peak of the freshwater HAB blooms in the St. Lucie Estuary (Fig. 4a).

### Indian River Lagoon

**Figure 7:**
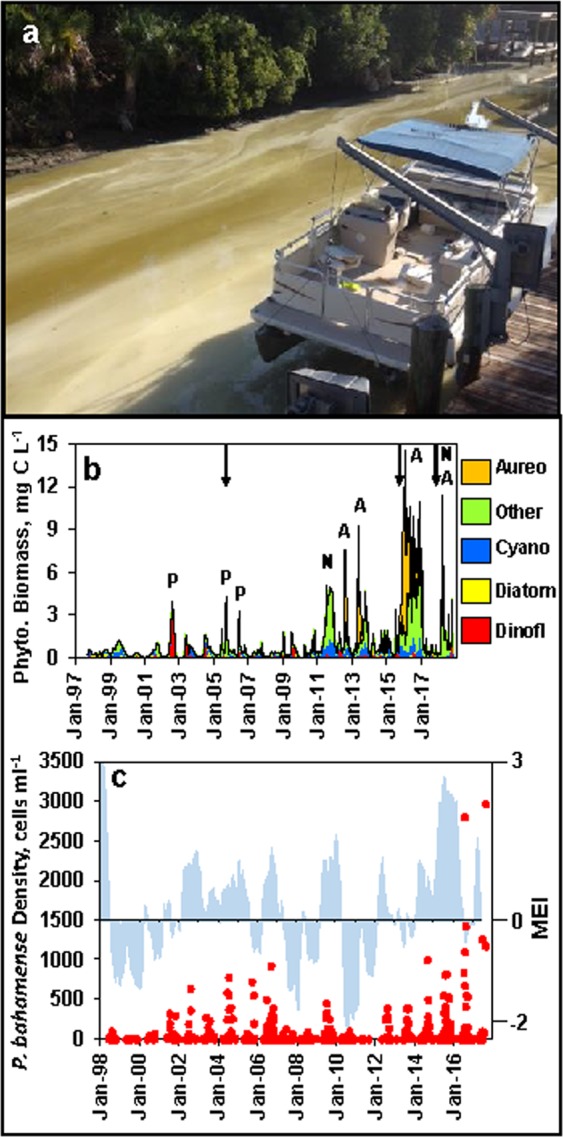
Brown tide event in the northern IRL dominated by *Aureoumbra lagunensis* (**a**, photo by permission from Kelly Young, Volusia County Environmental Management), and phytoplankton biomass for Site IRL1 in the IRL (**b**), divided into four major groupings, i.e. dinoflagellates, diatoms, cyanobacteria, *A. lagunensis* (Aureo) and all “other” taxa (letter above major bloom peaks indicate the dominant species in terms of biomass: i.e. A – *A. lagunensis*, N – unspecified nanoplanktonic eukaryotes, P – *P. bahamense*). Arrows show timing of hurricane/storm events. Panel ‘c’ *Pyrodinium bahamense* cell densities (red markers) and Multivariate ENSO Index (MEI) (blue) from 1998 to 2017 throughout the northern IRL.

**Figure 8:**
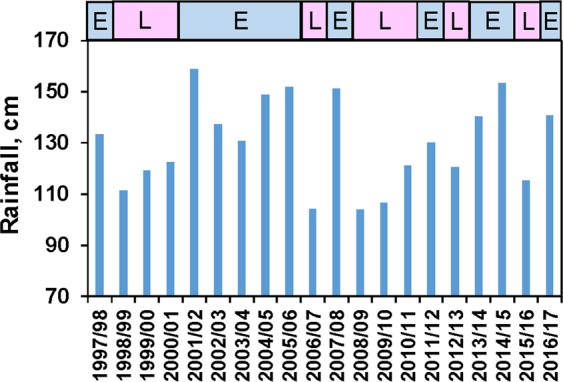
Annual rainfall totals at the NOAA meteorological station at Titusville, Florida, located near Site IRL1 in the northern IRL (www.ncdc.noaa.gov/IPS). The annual values are based on the 12-month period from November-October of each period. Letters in boxes above the figure represent time periods with predominantly El Niño (‘E’) or La Niña (‘L) conditions, based on Multivariate ENSO Index (MEI).

For the Indian River Lagoon ecosystem, a long-term continuous 20-year time-series of phytoplankton composition and biomass dating back to 1997 provides an opportunity not only to examine hurricane effects, but also more general trends in climatic effects on HABs. The northern Indian River Lagoon has repeatedly experienced intense HABs since 1997 (i.e. >2 µg carbon mL^−1^, or roughly equivalent to >30–50 µg chlorophyll a L^−1^) (e.g. Fig. 7a), as illustrated by the time-series at Site IRL1 (Figs. 1 and 7b, Supplemental Fig. S3). One of the trends in the time series is the positive relationship between rainfall and peaks in phytoplankton biomass^14,55^. The trend is indicated by the positive linear relationship between bloom biomass peaks of the toxic dinoflagellate *Pyrodinium bahamense* and rainfall prior to the blooms (R^2^ = 0.45, Supplemental Fig. S4). *P. bahamense* is one of the dominant bloom-forming HAB species in the Indian River Lagoon (Fig. 7b, Supplemental Fig. S3), and a major HAB species in other Florida ecosystems and many tropical ecosystems around the world^56,57^. One of the important cyclical phenomena that affects rainfall in central Florida is El Niño/La Niña periods. El Niño periods are often characterized by higher rainfall than La Niña periods (Fig. 8), particularly during the dry season (i.e. Nov.-April)^58,59^. A comparison of the temporal records of *P. bahamense* biomass and El Niño/La Niña periods (expressed as Multivariate ENSO Index: MEI) further demonstrates the relationship between peak *P. bahamense* biomass and high rainfall El Niño periods (Fig. 7c). The relationship is functionally tied to the positive relationship between nutrient concentrations and rainfall^14,56^. External nutrients enter the northern Indian River Lagoon from a variety of sources, including surface water runoff, groundwater discharge, direct rainfall inputs, septic system leakage, and permitted and accidental releases from sewage treatment systems^60–65^. All of these processes can be enhanced by high rainfall, although the relative importance of the sources can vary by nutrient type. For example, atmospheric contribution to non-point source nitrogen loads are significantly greater (i.e. 32–53%) than for phosphorus loads (i.e. 4–13%) (Gao 2009).

Beyond the general effects of elevated rainfall and nutrient loads on HAB potential, hurricanes can exacerbate the effect in several other ways, as observed in Lake Taihu, China^13^. Intense winds can impact nutrient concentrations through sediment re-suspension and re-mineralization of nutrients from damaged aquatic vegetation (e.g. seagrasses) and damaged terrestrial biomass in the watershed which can potentially be transported into the estuary. Because of long water residence times in the northern Indian River Lagoon (i.e. E_50_ of 100–200 days, 50% half water turnover rates) storm impacts can extend for months. For example, the effects of storm events in July-October (i.e. peak period for tropical activity) can have both short-term and long-term impacts, including elevated nutrient concentrations which can extend into the following year. The phytoplankton biomass time-series for Site 1 in the northern Indian River Lagoon provide an example of the latter phenomena (Fig. 7b. Supplemental Fig. S3). The tropical storm seasons of 2005, 2015 and 2017 all had storms with high rainfall totals^66^. All three years were associated with significant increases in nutrient concentrations (Supplemental Fig. S5) and HABs blooms in the following years, i.e. 2006, 2016 and 2018 (Fig. 7b, Supplemental Fig. S3). In 2006, the bloom involved the toxic dinoflagellate *P. bahamense*^14,56^. In 2016 and 2018, the bloom events also involved the brown tide species *Aureoumbra lagunensis*, as well as other nanoplanktonic eukaryotic algae (Fig. 7a,b, Supplemental Fig. S3)^66^.

The dramatically higher bloom biomass peaks in 2016 and 2018 relative to 2006 are the result of a shift in the intensity of blooms that began in the northern Indian River Lagoon in 2011^14^. The shift also involved significant changes in the structure of the ecosystem, such as widespread and major losses of seagrass communities, which may have intensified the response of the phytoplankton community to external and internal nutrient loads^14,66^. It is also possible that high winds associated with storms in 2015 and 2017 contributed to the persistence of the shift by disrupting the stability of surface sediment layers, and limiting seagrass recovery. The trend toward more frequent and intense blooms may be further accentuated if future storms and El Niño become more frequent and are associated with higher rainfall totals, as predicted by some climate models, which tie future increases in ocean water temperatures to increases in atmospheric water content^4–6,67,68^. In this context, the added dimension of temperature increases add up to a triple threat for bloom development, i.e. by enhancing nutrient loads, increasing algal growth rates and promoting dominance by cyanobacteria and other HAB species^1,3,68–71^.

### Direct versus indirect impacts

The two estuaries highlighted in this study illustrate how the impacts of hurricanes and El Niño periods on HABs not only depend on direct effects on nutrient loads that drive blooms, but also differences in the structure and function of individual ecosystems, such as water residence time, flushing rates, as well as indirect (i.e. allochthonous) introduction of blooms from the watershed. For ecosystems with long water residence times and shallow depths, like Lake Okeechobee and the northern Indian River Lagoon, enhanced watershed nutrient loads caused by hurricane and El Niño-related rainfall can directly enhance the potential for autochthonous HAB events. Physical disturbance of sediments by storm events can also enhance internal nutrient loads, as observed in Lake Taihu, China^13^. Similar observations have been made in Florida Bay, a restricted estuary on the southern tip of the Florida peninsula. Three hurricanes impacted Florida Bay in 2005 (i.e. Katrina, Rita and Wilma), resulting in transport of nutrient rich sediments into the eastern bay, increased nutrient load from the bay’s watersheds and destruction of mangrove habitat. The hurricane period was followed by intense marine picoplanktonic cyanobacteria blooms from 2005–2008, in part aided by the very long water residence times in the bay^31,72,73^.

By contrast, the St. Lucie Estuary presents a different picture, in part because of the shorter and more variable water residence times (i.e. 1–16.5 days)^19,39^, and the linkage to bloom-prone Lake Okeechobee. As a result, the greatest potential for autochthonous marine algal blooms occurs during periods of comparatively low rainfall, watershed discharge and nutrient levels, but longer water residence times which permit the accumulation of phytoplankton biomass. Conversely, periods of high rainfall, watershed discharge and nutrient inputs from the watershed can be associated with lower peak phytoplankton biomass levels due to short water residence times. Similar relationships have been observed in two other ecosystems in Florida, the Guana, Tolomato, Matanzas estuary^41,74,75^ and Lake George^17,76^. In both ecosystems the strong hurricane seasons of 2004 and 2005 yielded reduced peaks in phytoplankton biomass due to reduced water residence times, despite elevated concentrations of nutrients. The exception to this trend in the St. Lucie Estuary is high rainfall periods associated with high discharges from Lake Okeechobee during intense freshwater HAB events in the lake, leading to freshwater HABs of allochthonous origin in the estuary^18,39^. Similar relationships have been observed in other estuaries^77^, such as the Caloosahatchee estuary in Florida^78^ and San Francisco Bay^79^.

Potential impacts of hurricanes and El Niño periods on HABs are not limited to coastal estuaries and inland lakes, but can extend into nearshore and open ocean environments, particularly in shallow shelf regions. The potential is illustrated by the intense red tide event experienced along the southwest coast of Florida in the summer of 2018^77,80–82^. The toxic dinoflagellate bloom, dominated by *Karenia brevi*s, extended over a broad reach of the inner shelf near several major freshwater outflows from the watershed, including large inputs from the Caloosahatchee River. The red tide resulted in serious impacts to aquatic animal and human health, as described for earlier red tide events in the region, including mass mortalities of marine animals and human health impacts related to exposure to aerosolized neurotoxins produced by *K. brevis* (i.e. brevetoxin)^81,83^. As in the case of the St. Lucie estuary, the Caloosahatchee River was subject to large discharges from Lake Okeechobee in response to the strong hurricane season in 2017 and high rainfall in the spring of 2018. Recent research has shown that periods of high discharge result in significant elevation of nutrient levels in the estuary^82^. It may be hypothesized that such discharges contribute to the nutrient supplies that support red tide events, such as the event observed in 2018^82^, highlighting the need for further research on land-sea interactions in relationship to coastal blooms.

Irrespective of the origin of HAB events, they can be disruptive to ecosystem structure and function in many ways, including production of toxins, promotion of hypoxic conditions, shading out of benthic primary producers (e.g. seagrasses) and alteration of food web dynamics^84^. Depending on the species involved, HABs can also pose threats to human and animal health, particularly as it relates to toxin exposure^52,53,81,83,85^. Beyond these direct harmful effects, there are indirect side effects to HAB phenomena, which can have important economic and life-style consequences^86,87^. Except for some inquisitive and committed phycologists, most people find the types of intense algal scums encountered in the St. Lucie Estuary (Figs. 2a and 5) and Indian River Lagoon (Fig. 7a) disturbing and undesirable, leading to impacts on tourism, recreational use and property values. In a sense, the visual imagery of these blooms sends a strong message on the sensitivity of aquatic ecosystems to changes in the environment related to human activities, including cultural eutrophication, hydrological alteration and climate change. These are multi-dimensional problems requiring multi-dimensional solutions based on a clear ecosystem-specific understanding of driving factors and consequences of HABs. The results of this study highlight the important roles that both stochastic (e.g. hurricanes and storms) and cyclical (e.g. El Niño/La Niña patterns) climatic processes can play in HAB dynamics. The anticipated future progressive changes in cultural eutrophication and global climatic conditions, if left unaddressed, will likely exacerbate existing weather-driven HAB instigations. The ecosystems included in this study are exemplary of many subtropical environments which are sensitive to climate changes due to their position in the transition between temperate and tropical environments, as well as high frequency of exposure to tropical storms. Many sub-tropical/tropical regions around the world are also subject to rapid population growth and development, heightening the challenges associated with cultural eutrophication.

## Supplementary information

Dataset 1.

## Data Availability

The data used in this paper were obtained from the South Florida Water Management District for Lake Okeechobee. Nutrient data were obtained from St. Johns River Water Management District for the Indian River Lagoon (Palatka, Florida), as part of project data reporting requirements and should be accessible from the respective Districts.
