# Supplementary material for: Hurricanes, El Niño and harmful algal blooms in two sub-tropical Florida estuaries: Direct and indirect impacts
Source: Sci Rep. 2020 Feb 5;10:1910. doi: 10.1038/s41598-020-58771-4 (PMC7002698; doi:10.1038/s41598-020-58771-4)
Supplement: Supplementary file 1 — Dataset 1. [file 41598_2020_58771_MOESM1_ESM.docx]

**Hurricanes, El Nińo and harmful algal blooms in two sub-tropical Florida estuaries: Direct and indirect impacts**

Phlips, Edward J., Susan Badylak, Natalie Nelson and Karl Havens

Supplemental figures

Fig. S1. Phytoplankton biomass (as carbon) at Sites 1 (North Fork) divided into four major groupings, i.e. dinoflagellates, diatoms, cyanobacteria and “other” (all other taxa). Letters above major bloom peaks (i.e. biomass > 2 µg carbon ml^-1^) indicate dominant species in terms of biomass, i.e. the marine dinoflagellates A - *Akashiwo sanguinea.*

Fig. S2. GIS image of the distribution of bloom biomass (as average chlorophyll *a* in the water column) in the St. Lucie estuary on August 12, 2005 (After Phlips et al. 2012).

Fig. S3. Phytoplankton biomass (as carbon) time series for IRL2 in the Banana River region of the Indian River Lagoon, divided into four major groupings, i.e. dinoflagellates, diatoms, cyanobacteria, *A. lagunensis* (Aureo) and “other” (all other taxa). Markers “▲” show timing of hurricane/storm events. Letter codes above major bloom peaks indicate the dominant species in terms of biomass: i.e. Al – *A. lagunensis*, Cy – picoplanktonic cyanobacteria, D – diatoms, N – unspecified nanoplanktonic eukaryotes, P – *P. bahamense*.

Fig. S4. Peak *Pyrodinium bahamense* biomass per year versus rainfall for two-month period before peak (After Phlips et al. 2015).

Fig. S5. Total nitrogen (TKN) and total phosphorus (TP) concentrations at Sites IRL1 and IRL2 in the Indian River Lagoon. Markers “▲” show timing of hurricane/storm events (Data from St. Johns River Water Management District, Palatka, Florida).
